# Microscopic white matter changes in the cingulum contribute to memory impairment among older adults with obstructive sleep apnea in the memory clinic

**DOI:** 10.1002/alz.71197

**Published:** 2026-02-13

**Authors:** Aaron Lam, Hannes Almgren, Jake Palmer, Angela L D'Rozario, Arkiev D'Souza, Brendon J Yee, Loren Mowszowski, Fernando Calamante, Sharon L Naismith

**Affiliations:** ^1^ The Healthy Brain Ageing Program, Brain and Mind Centre The University of Sydney Camperdown New South Wales Australia; ^2^ School of Psychology Faculty of Science The University of Sydney Camperdown New South Wales Australia; ^3^ The Woolcock Institute of Medical Research Macquarie Park New South Wales Australia; ^4^ School of Biomedical Engineering, Faculty of Engineering The University of Sydney Camperdown New South Wales Australia; ^5^ School of Psychological Sciences Faculty of Medicine, Health and Human Sciences Macquarie University Macquarie Park New South Wales Australia; ^6^ Royal Prince Alfred Hospital, Central Clinical School Sydney University Sydney New South Wales Australia; ^7^ Macquarie Medical School Macquarie University Macquarie Park New South Wales Australia; ^8^ Charles Perkins Centre The University of Sydney Camperdown New South Wales Australia

**Keywords:** ageing, alzheimer's disease, dementia, learning, memory, memory clinic, sleep apnea, white matter tracts

## Abstract

**INTRODUCTION:**

Obstructive sleep apnea (OSA) is prevalent in memory clinic patients and is associated with learning and memory deficits. In a memory clinic sample, we investigated the relationship between memory‐related white matter pathways and OSA.

**METHODS:**

Eighty‐two participants (mean age 67.0) underwent neuropsychological testing and neuroimaging. Fixel‐based white matter analyses were conducted in the anterior thalamic radiation (ATR), cingulum, uncinate fasciculus (UF), and fornix. Oxygen desaturation index (ODI) from overnight polysomnography classified participants as controls (ODI < 5, *n* = 26), mild OSA (ODI 5–14, *n* = 32), or moderate and severe OSA (ODI ≥ 15, *n* = 24).

**RESULTS:**

In mild OSA, white matter changes were seen in the ATR, UF, and fornix. In moderate and severe OSA, alterations were observed in the cingulum and fornix. Cingulum changes were linked to poorer verbal learning and memory.

**DISCUSSION:**

OSA is associated with disrupted memory‐related pathways. Cingulum changes are associated with memory performance in moderate and severe cases.

## Highlights

- Fixel‐based white matter analysis in older adults attending memory clinics revealed widespread microstructure alterations in mild obstructive sleep apnea (OSA), particularly in the anterior thalamic radiation, uncinate fasciculus, and fornix.
- Moderate and severe OSA were associated with white matter alterations predominantly in the cingulum and fornix.
- Cingulum alterations were associated with poorer verbal learning and memory, independent of hippocampal volume.
- Findings suggest a novel mechanistic link between OSA‐related white matter changes and cognitive impairment in older adults with subjective cognitive impairment and mild cognitive impairment.

## 1 BACKGROUND

Within the older community and memory clinic samples, obstructive sleep apnea (OSA) is evident in up to 70% of patients.^1^ It has been linked to memory impairment,^2^ hippocampal volume loss,^3^ functional uncoupling within the brain,^4^ and dementia.^5^, ^6^ Identifying modifiable risk factors such as OSA and understanding how they affect brain networks that support memory is essential for informing intervention strategies aimed at secondary dementia prevention.^7^

Two populations at risk for dementia are individuals with subjective cognitive impairment (SCI) and mild cognitive impairment (MCI). SCI reflects self‐reported cognitive issues without objective neuropsychological deficits; however, those with SCI are at an increased risk of developing dementia, particularly those in a memory clinic cohort.^8^ Those with MCI exhibit neuropsychological decrements of ≥ 1.5 standard deviations below normative data,^9^ and around 45% will convert to dementia over five years,^10^ with the amnestic subtype at particularly high risk of developing Alzheimer's disease (AD).^11^ In contrast, non‐amnestic MCI presentations are more susceptible to non‐Alzheimer's dementias.^12^

White matter tracts that support memory, particularly those connecting to the medial temporal lobe, are affected early in the pathogenesis of AD and may be especially vulnerable to OSA‐related changes. These include the anterior thalamic radiation (ATR), cingulum, uncinate fasciculus (UF), and fornix.^13^, ^14^, ^15^, ^16^, ^17^ Nonetheless, the interrelationship between specific tract changes, OSA severity, and memory performance remains underexplored in at risk individuals such as those with SCI and MCI.

Few studies have used diffusion tensor imaging (DTI) to examine OSA in older adults, with most focusing on middle‐aged samples. Baril et al.^18^ reported reductions in mean diffusivity (MD), axial diffusivity (AD), and radial diffusivity (RD) but no significant differences in fractional anisotropy (FA) in older adults with mild OSA, compared to controls, using tract‐based spatial statistics. Notably, many of these effects were not observed in the moderate and severe OSA group. Conversely, Lee et al.^19^ used tract metrics to demonstrate FA reductions in the anterior cingulum and frontal regions in older adults with mild and moderate OSA, compared to those without OSA. Similarly, in older adults with MCI, Marchi et al.^20^ showed that nocturnal intermittent hypoxemia altered the fornix, based on conventional diffusion metrics (MD, AD, RD, and FA). Altered fornix microstructure was associated with poorer memory performance. While these findings underscore the utility of diffusion imaging, conventional tensor measures (i.e., DTI) often lack specificity, especially in regions with complex fiber architecture (e.g., crossing fibers), which constitute up to 90% of white matter.^21^ An emerging alternative, fixel‐based analysis (FBA), separately examines fiber populations within a voxel, addresses this limitation by quantifying fiber density (FD), fiber‐bundle cross‐section (FC), and fiber density and cross‐section (FDC).^22^ These metrics reflect intra‐axonal volume and bundle morphology, offering greater sensitivity to microstructural changes such as axonal loss and fiber degeneration.^22^ Hence, employing FBA to investigate OSA's impact on white matter could yield more profound insights into the microstructural integrity and vulnerability of white matter pathways in early disease stages.

Discrepancies in prior studies may be due to population differences that lead to inconsistent findings; for example, Marchi et al. found significant links between hypoxemia and the fornix only in MCI patients, not in controls.^20^ Methodological limitations exacerbate this. As previously mentioned, traditional tract‐based spatial statistics and DTI methods may not capture subtle and early white matter changes.^23^

This study compared white matter changes in older adults from a memory clinic with no, mild and moderate and severe OSA, focusing on memory‐critical tracts: ATR, cingulum, UF, and fornix. We aimed to determine whether these alterations were linked to learning and memory performance, and whether these relationships are independent of hippocampal volume, given the hippocampus's well‐established role in learning and memory. We hypothesized that altered fixel‐based metrics in the ATR, cingulum, UF, and fornix would be most pronounced in the moderate and severe group, followed by the mild group, compared to the control group. Furthermore, we posited that they would be associated with poorer learning and memory performance in participants with OSA.

**RESEARCH IN CONTEXT**

1. **Systematic review**: We reviewed the existing literature using PubMed, Google Scholar, and recent conference proceedings to identify studies examining white matter alterations associated with obstructive sleep apnea (OSA) and their cognitive correlates in older adults. Prior studies have reported diffuse white matter microstructural alterations in OSA; however, few have focused on older adults with subjective or mild cognitive impairment (MCI) attending memory clinics. To date, no studies have applied advanced fixel‐based analysis to disentangle fiber‐specific white matter pathology or to determine whether these alterations contribute to learning and memory impairment in this population.
2. **Interpretation**: Our findings demonstrate that mild OSA is associated with widespread white matter alterations, while moderate and severe OSA primarily affects the cingulum and fornix. Cingulum integrity was linked to poorer verbal learning and memory, independent of hippocampal volume, suggesting a novel mechanistic link between OSA‐related white matter disruption and cognitive impairment.
3. **Future directions**: Longitudinal studies are necessary to determine whether OSA‐related white matter changes predict cognitive decline and to assess whether treating OSA can mitigate white matter injury and cognitive deterioration.

## 2 METHODS

### 2.1 Participants

Participants aged ≥ 50 with cognitive and/or mood concerns were recruited through the Healthy Brain Ageing Clinic, University of Sydney. Participants sought assessment at the memory clinic for recent cognitive changes rather than sleep‐related symptoms. All assessments required a general practitioner (GP) or specialist referral, and participants were screened initially for the following exclusion criteria: primary language other than English; a diagnosis of dementia or Mini‐Mental State Examination (MMSE) less than 24; significant neurological or psychiatric disorder (e.g., Parkinson's disease, epilepsy, schizophrenia); previous stroke; history of significant substance abuse (including alcohol abuse defined by > 14 standard drinks per week); intellectual disability; and, history of traumatic brain injury with a loss of consciousness greater than 30 min. For this sub‐study, participants were required to have undergone a polysomnography (PSG) and a magnetic resonance imagin (MRI) scan within a 180‐day time frame (median = 7.0 days, average = 20.6 days). Furthermore, individuals with a current diagnosis of OSA who were receiving treatment (e.g., continuous positive airway pressure, mandibular advancement device, or other therapies) were excluded from this analysis. The study was approved by The University of Sydney Human Research Ethics Committee (No. 2019/HE000271), and all participants provided written informed consent.

### 2.2 Clinical assessment

As detailed previously,^24^ all participants underwent comprehensive medical, neuropsychological, and psychological assessments. A specialist conducted a semi‐structured interview to gather medical, mood and sleep history information, and completed the Cumulative Illness Rating Scale Geriatric (CIRS‐G).^25^ The CIRS‐G measured medical illness burden, where a higher score indicated a more significant medical illness burden. Body mass index (BMI), antidepressant use, hypnotic medication use, antihypertensive use, and weekly alcohol consumption were collected and reported for descriptive purposes.

While a research psychologist used a semi‐structured interview to assess lifetime and current depression and anxiety, for this study, we included scores on the 15‐item Geriatric Depression Scale (GDS‐15)^26^ for descriptive purposes. Higher scores indicated more severe depressive symptoms (scores ranged 0–15).

Lastly, participants self‐reported sleep quality using the Pittsburgh Sleep Quality Index for descriptive purposes. Higher scores indicated poorer sleep quality (scores ranged 0–21).^27^

### 2.3 Neuropsychological assessment

A clinical neuropsychologist administered a standardized test battery covering multiple domains of cognition, as previously described.^28^ For this study, we examined verbal learning and memory using the Rey Auditory Verbal Learning Test (RAVLT).^29^ Verbal learning was calculated by the sum of the first five trials, while memory was calculated from the delayed recall component (trial 7). Age and education adjusted *z*‐scores based on normative data^30^ were utilized.

For descriptive purposes, the MMSE^31^ and the Wechsler Test of Adult Reading (WTAR)^32^ are also reported. The MMSE is a widely used cognitive screening tool. Scores ranged from 0 to 30, where lower scores indicated poorer performance (the generally accepted cut‐off for dementia is < 24). The WTAR measured premorbid intelligence, where higher scores indicated a higher predicted intelligence quotient.

Following the clinical assessment, MCI was classified via consensus between the medical specialist and two neuropsychologists, following established criteria,^33^ which required decrements of ≥ 1.5 standard deviations (below age and education‐adjusted premorbid estimates) on neuropsychological tests. Participants who did not meet MCI criteria were classified as SCI due to subjective cognitive concerns.

### 2.4 PSG

PSG was recorded using Alice 5, Embla Titanium, and ProFusion 4. The following electroencephalography (EEG) signals were derived: F3, F4, C3, C4, O1, O2, with contralateral reference to the mastoids, as well as Fz, Cz, and Pz, which were referenced to the average of the mastoids. Signals were sampled at 200 Hz (Alice 5) or 512 Hz (Embla Titanium and Profusion 4). Sleep stages and respiratory events were scored by a trained sleep technician using standardized AASM 2.3 criteria.^34^ The following sleep measures were reported for descriptive purposes: Apnea‐Hypopnea Index (AHI), 3% oxygen desaturation index (ODI), total sleep time, sleep efficiency (total sleep time divided by total time spent in bed), wake after sleep onset (WASO; the amount of time spent awake after first epoch (30 s) of sleep, amount of non‐rapid eye movement (NREM) sleep, and amount of REM sleep. Because our primary interest was nocturnal hypoxemia and its potential contribution to white matter injury, we classified OSA severity using the ODI, which more directly reflects intermittent nocturnal hypoxemia than the AHI, and is widely validated as a marker of disease burden. Participants were divided into three groups based on the severity of OSA: non‐OSA (control group, ODI < 5), mild OSA (ODI 5–14.9), and moderate and severe OSA (ODI ≥ 15).

### 2.5 Neuroimaging

#### 2.5.1 MRI acquisition

All participants underwent an MRI scan using a 3T Discovery MR750 (GE Medical Systems, Milwaukee, WI, USA) scanner with an eight‐channel phased‐array head coil at the Brain and Mind Centre, University of Sydney. A 3D‐T1‐weighted structural image was collected using a BRAVO Spoiled Gradient‐Recalled (SPGR) sequence with 196 sagittal slices using the following parameters: repetition time = 7.3 ms, echo time = 2.8 ms, flip angle = 12°, acquisition matrix = 256×256 with 0.9 mm isotropic voxels. The protocol also included diffusion‐weighted imaging, which was acquired with a repetition time = 8,000 ms, echo time = 84 ms, 64 slices of 2.5 mm thickness and 32 gradient directions (*b* = 1,000 s/mm^2^); two reference images without diffusion‐weighting were also collected (*b* = 0 s/mm^2^).

#### 2.5.2 Diffusion MRI processing and analysis

##### MRI Preprocessing

All diffusion MRI analyses were performed using *MRtrix3* (version 3.0.2).^35^ Preprocessing was in line with recent recommendations,^22^ and included distortion correction, motion correction, and bias field correction.^21^, ^36^, ^37^, ^38^, ^39^

##### Fiber orientation distribution

Fiber orientation distributions (FODs)^40^ were estimated following Dhollander et al.^22^ For fixel‐based analyses (where “fixel” refers to a fiber population within a voxel), a group‐averaged FOD template was created incorporating 10 representative subjects from each group. Each subject's intensity‐normalized FOD image was then aligned to this study‐specific template.

##### White matter tract segmentation

TractSeg was used to automatically segment white matter tract bundles (TractSeg version 2.2,^41^ https://github.com/MIC‐DKFZ/TractSeg) directly from the FODs using a convolutional neural network. The following bilateral bundles were considered in our study: fornix, ATR, UF, and cingulum. Each tract was visually inspected for segmentation quality.

##### Fixel‐based metrics

FD, which reflects the density of fiber bundles (i.e. a microstructure measure); FC, which quantifies the cross‐sectional area of fiber bundles (i.e. a macrostructure measure); and FDC, a combined metric, were calculated as outlined in Dhollander et al.^22^

##### Hippocampal volume

Hippocampal volumetrics were calculated using the T1‐weighted image with recon‐all from FreeSurfer (v6.0).^42^ Hippocampal volume was corrected for estimated intracranial volume (eICV) and reported as a percentage ([hippocampal volume/eICV]×100). Hippocampal segmentations were visually inspected to ensure segmentation quality. Total hippocampal volume was calculated as the sum of the left and right hemispheres.

### 2.6 Statistical analyses

The normality of demographic, clinical, memory, and sleep data values was evaluated using the Kolmogorov‐Smirnov test and a visual inspection of frequency distributions and Q‐Q plots. Log transformations were implemented to correct for non‐normally distributed variables when applicable. Group differences in demographic and baseline sleep characteristics were assessed using one‐way analysis of variance (ANOVA) for variables that were approximately normally distributed, applying log transformations as necessary. When the assumptions of normality were not met, and transformations were insufficient, non‐parametric Kruskal‐Wallis tests were utilized. Tukey's honest significant difference (HSD) was conducted for post hoc pairwise comparisons when the data was normally distributed, while the Wilcoxon test was applied for non‐normally distributed data. Significance was set at *p* < 0.05.

Fixel‐based statistical analyses were conducted using *MRtrix3*. General linear models (GLMs) using nonparametric permutation tests were applied using *fixelcfestats* to assess significant group differences (control vs. mild OSA and vs. moderate and severe OSA) in FD, FC, and FDC in each tract of interest. FBA uses a template tractogram for connectivity‐based statistical fixel enhancement.^43^ These streamlines were filtered to 2 M using the SIFT algorithm.^44^ Multiple comparisons were controlled using family‐wise error (FWE) correction, and a significant alpha level was set at 0.05. Additional exploratory analyses were conducted to control for other vascular risk factors including BMI and hypertension. For white matter tracts that showed significant differences between groups, within‐group analyses were conducted to examine associations with verbal learning and memory performance while controlling for hippocampal volume.

If the white matter fiber tract is significantly associated with learning and memory within groups, we conducted further linear regression analyses to confirm whether the relationship between white matter changes and cognitive function was anatomically specific to the region of the tract identified through the between‐group comparison. First, we extracted the average fixel‐based metric from the tract segment that demonstrated a significant difference between control participants and the exposure group (mild or moderate and severe OSA). An FWE‐corrected mask was created, which contained fixels that were significantly different between groups and used for data extraction. Mean fixel‐based metrics from each mask were computed for each participant. Subsequently, linear regression models were conducted within the exposure group, using the average fixel‐based metric as the predictor variable and the verbal learning or memory performance as the dependent outcome. As a supplementary exploratory analysis, we repeated the linear regression model within the control group. Statistical significance was set at *α* = 0.05 for all regression analyses.

## 3 RESULTS

A total of 82 participants were included in this analysis, consisting of 26 controls (without OSA), 32 with mild OSA, and 24 with moderate and severe OSA. For cognitive classifications, 23 participants had SCI and 59 had MCI. Among them, there were 50 females and 32 males. The average age of the participants was 67.0 years, with 13.3 years of education. In terms of BMI, participants were, on average, classified as overweight (BMI = 27.4). Additionally, participants reported a low level of depressive symptoms (mean = 3.6) and self‐reported poor sleep quality (mean = 6.7).

The complete group comparisons between OSA groups regarding demographics and sleep are presented in Table 1. No differences were found between OSA groups in terms of age, sex, years of education, global cognition (MMSE), premorbid IQ (WTAR), or depressive symptoms (GDS‐15) (*p* > 0.05). Although the overall ANOVA indicated a significant group difference in CIRS‐G scores, post hoc pairwise comparisons did not reveal any statistically significant differences between groups. Participants with mild or moderate and severe OSA had significantly higher BMI than those without OSA (*p* = 0.002).

**TABLE 1:** Demographic, clinical, and sleep information for whole sample, and within control and OSA groups.

Parameter	Control *n* = 26	Mild OSA *n* = 32	Moderate and severe OSA *n* = 24	Test statistic	*p*‐Value
Age, years	64.7 ± 8.5	68.4 ± 8.7	67.5 ± 7.8	1.41	0.238
Sex, female count (%)	16 (62%)	22 (69%)	12 (50%)	2.03	0.362
Education, years	13.7 ± 3.2	14.0 ± 2.5	12.1 ± 3.1	3.47	0.066
MMSE^a^	29.0 ± 2.0	29.0 ± 1.8	29.0 ± 2.0	0.35	0.839
Premorbid IQ^a^	110.5 ± 9.5	110.5 ± 11.3	106.0 ± 7.0	5.46	0.065
GDS‐15^a^	2.5 ± 3.8	2.5 ± 3.0	3.0 ± 6.3	0.49	0.781
CIRS‐G	3.5 ± 3.0	3.0 ± 4.0	5.0 ± 4.0	4.19	0.045^*^
Learning, *z*‐score	−0.03 ± 1.3	−0.46 ± 1.0	0.11 ± 0.7	0.14	0.709
Memory, *z*‐score	−0.05 ± 1.5	−0.37 ± 1.0	0.26 ± 0.8	0.63	0.430
BMI, kg/m^2^	24.8 ± 3.4	27.01 ± 8.7	30.59 ± 4.4	10.29	0.002^**^
Hypertension, yes (%)	8 (32%)	11 (34%)	15 (63%)	5.92	0.052
Sleep quality	6.7 ± 2.8	7.1 ± 4.2	6.2 ± 3.4	0.17	0.679
Total sleep time, mins	329.0 ± 56.9	358.8 ± 40.8	325.2 ± 77.4	0.06	0.809
WASO, mins	87.9 ± 53.6	76.6 ± 40.6	99.0 ± 53.23	0.62	0.435
Sleep efficiency, %^a^	77.6 ± 17.3	79.0 ± 11.8	76.0 ± 14.2	−1.83	0.400
Sleep latency, min^b^	20.1 ± 26.9	20.5 ± 22.6	28.3 ± 41.6	0.58	0.449
NREM duration, min^a^	245.3 ± 31.0	286.5 ± 54.0	276.0 ± 35.2	9.30	0.009^**^
REM duration, min	76.0 ± 34.8	68.9 ± 20.9	50.8 ± 28.5	9.37	0.003^**^
AHI, per hour^b^	7.7 ± 8.3	12.0 ± 7.6	36.8 ± 23.7	56.93	0.001^**^
ODI, per hour^b^	2.4 ± 1.3	7.9 ± 1.8	35.2 ± 21.1	461.80	0.001^**^
Arousal index, per hour^b^	15.9 ± 12.0	16.1 ± 9.1	28.1 ± 17.0	7.45	0.008^**^

*Note*: Data are presented as mean and standard deviation, unless stated otherwise. Group comparisons were conducted with independent student t‐test unless stated otherwise. Pairwise comparisons: CIRS‐G = no significant pairwise comparisons; BMI = control > mild = moderate and severe; NREM duration = control < mild = moderate and severe; REM duration = moderate and severe < control = mild; AHI = control < mild < moderate and severe; ODI = mild; AHI = control < mild < moderate and severe.

Abbreviations: AHI, Apnea‐Hypopnea Index; BMI, body mass index; CIRS‐G, Cumulative Illness Rating Scale—Geriatric version; GDS‐15, 15 item Geriatric Depression Scale‐15 items; IQ, intelligence quotient; MMSE, Mini‐Mental State Examination; NREM, non‐rapid eye movement; ODI, oxygen desaturation index; OSA, obstructive sleep apnea; REM, rapid eye movement; WASO, wake after sleep onset.

* *p* < 0.05.

** *p* < 0.01.

^a^ Median and interquartile range is presented, and Kruskal‐Wallis was used to compare group differences. Pairwise comparisons: CIRS‐G = no significant pairwise comparisons

^b^ Log transformed was used for group comparisons but untransformed mean and standard deviation are presented

Regarding sleep, pairwise comparisons indicated that both AHI and ODI were the highest in the moderate and severe OSA group, followed by the mild OSA group, with the control group having the lowest values (*p* = 0.001 for all pairwise comparisons). Furthermore, individuals with mild OSA exhibited reduced NREM sleep duration compared to controls (*p* = 0.004), but did not differ significantly from those with moderate and severe OSA (*p* = 0.026). No differences in NREM sleep duration were noted between the control and moderate and severe OSA groups (*p* > 0.05). In terms of REM duration, there was no difference between controls and mild OSA (*p* > 0.05). However, the moderate and severe OSA group had lower REM duration than the control group (*p* = 0.038) and the mild OSA group (*p* = 0.003). Lastly, there were no differences in total sleep time, wake after sleep onset, sleep efficiency, or sleep latency (*p* > 0.05).

### 3.1 Between‐group FBA comparison between control versus mild OSA

**FIGURE 1:**
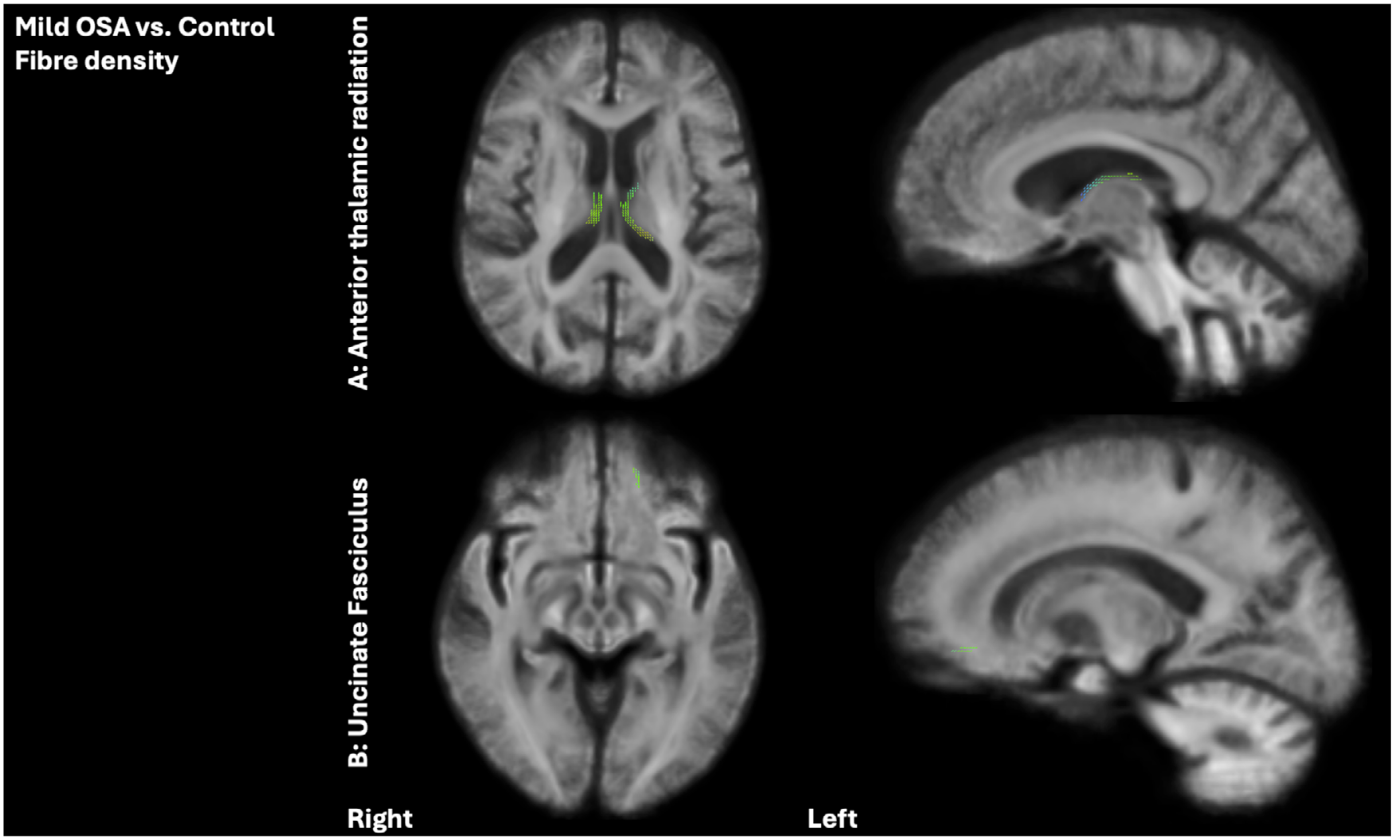
Fixel‐based comparison of white matter alterations between mild OSA and control groups in older adults at‐risk of dementia, showing the significant fixels after FEW correction (*p* < 0.05). Results of fixel‐wise generalized linear models showing a decrease in fiber density within the ATR and uncinate fasciculus tracts in mild OSA, compared to the control group. Each fixel is color‐coded according to its principal fiber orientation: Red represents left‐right (*x*‐axis), green represents posterior‐anterior (*y*‐axis), and blue represents inferior‐superior (*z*‐axis) directions. ATR, anterior thalamic radiation; OSA, obstructive sleep apnea.

**FIGURE 2:**
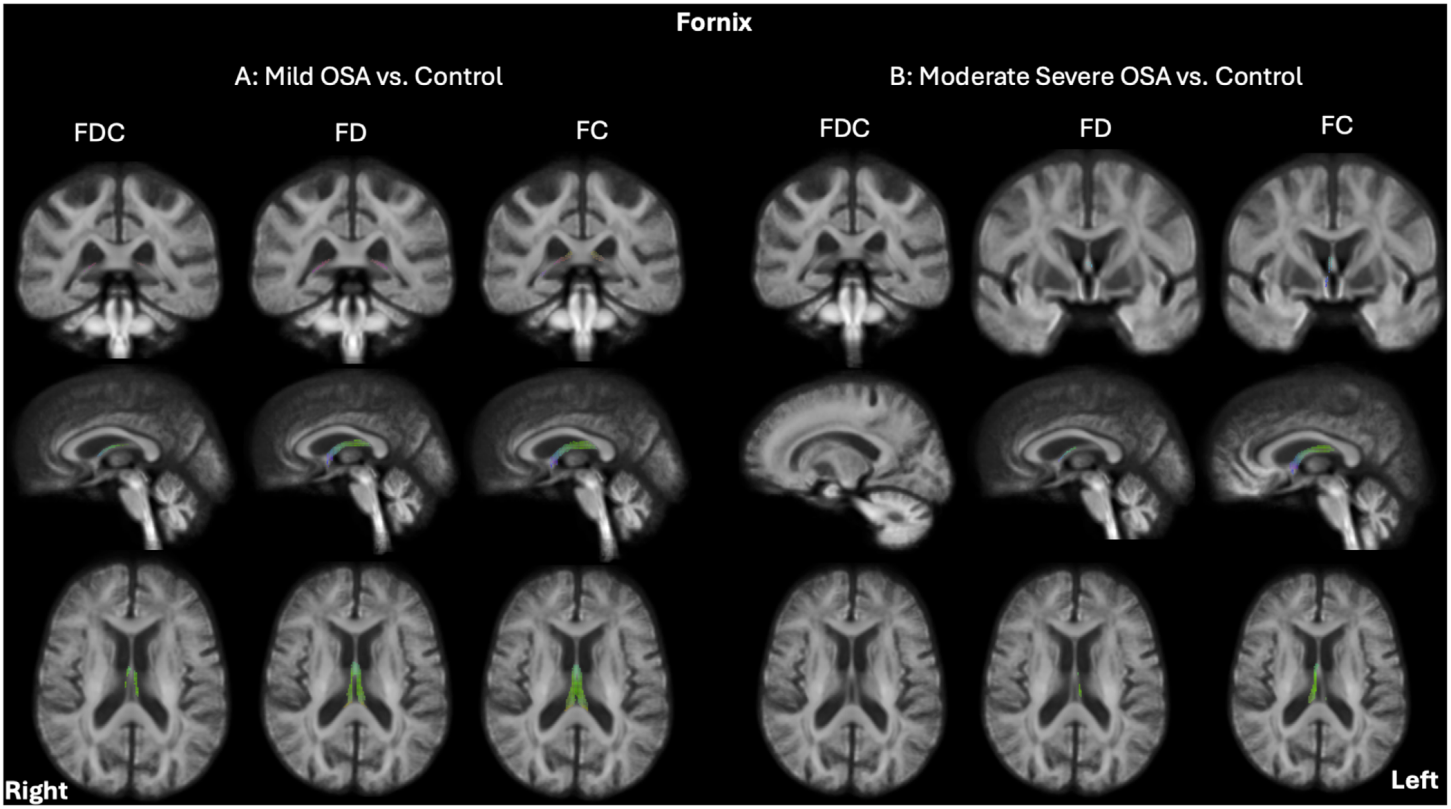
FBA of white matter alterations in the fornix comparing older adults at‐risk of dementia with (A) mild OSA and (B) moderate and severe OSA to the controls, showing the significant fixels after FEW correction (*p* < 0.05). (A) Results of fixel‐wise generalized linear models showing a decrease in FDC, FD, and increase in log FC within the fornix tract in mild OSA, compared to the control group. (B) Results of fixel‐wise generalized linear models showing a decrease in FD and increase in log FC within the fornix tract in moderate and severe OSA, compared to the control group. The FDC metric was not significantly different between the moderate and severe OSA and control groups. Each fixel is color‐coded according to its principal fiber orientation: Red represents left‐right (*x*‐axis), green represents posterior‐anterior (*y*‐axis), and blue represents inferior‐superior (*z*‐axis) directions. FC, fiber cross‐section; FD, fiber density; FDC, fiber density cross‐section; OSA, obstructive sleep apnea.

Compared to the control group, individuals with mild OSA exhibited significant reductions in FDC within the left ATR (number of significant fixels: *n* = 3) and UF (number of significant fixels: *n* = 21), as well as reductions in the FD (as illustrated in Figure 1) within the bilateral ATR (309 and 235 significant fixels in the left and right, respectively) and the left UF (57 fixels). Moreover, individuals with mild OSA demonstrated a widespread and significant decrease in both FDC (138 and 97 significant fixels in the left and right, respectively) and FD (1004 and 918 significant fixels in the left and right, respectively), alongside an increase in log FC (1074 and 1144 significant fixels in the left and right, respectively) within the fornix compared to the control group (depicted in Figure 2A).

After controlling for BMI, there was a bilateral alteration in FDC for bilateral ATR (number of significant fixels: *n* = 1 for left and right) and left UF (number of significant fixels = 2). Similarly, the reduced FD for the bilateral ATR (454 and 286 significant fixels in the left and right, respectively) and left UF survived correction for BMI. The left fornix remained significant after adjustment for BMI, but not the right fornix. More specifically, there was a decrease in FD (number of significant fixels: 1180) and FDC (number of significant fixels: 269), as well as an increase in log FC (number of significant fixels: 1276).

After controlling for hypertension, the reduction in FDC in the left ATR and left UF remained significant (2 and 10 significant fixels, respectively). The reduced FD also remained significant in the bilateral ATR (321 and 206 significant fixels in the left and right, respectively) and the left UF (number of significant fixels: 52). When correcting for hypertension, the bilateral fornix survived corrections for all fixel measures. More specifically, reduced FD (935 and 859 significant fixels in the left and right, respectively) and FDC (83 and 103 significant fixels in the left and right, respectively), and increase in log FC (1002 and 1116 significant fixels in the left and right, respectively).

### 3.2 Between‐group FBA comparison between control versus moderate and severe OSA

**FIGURE 3:**
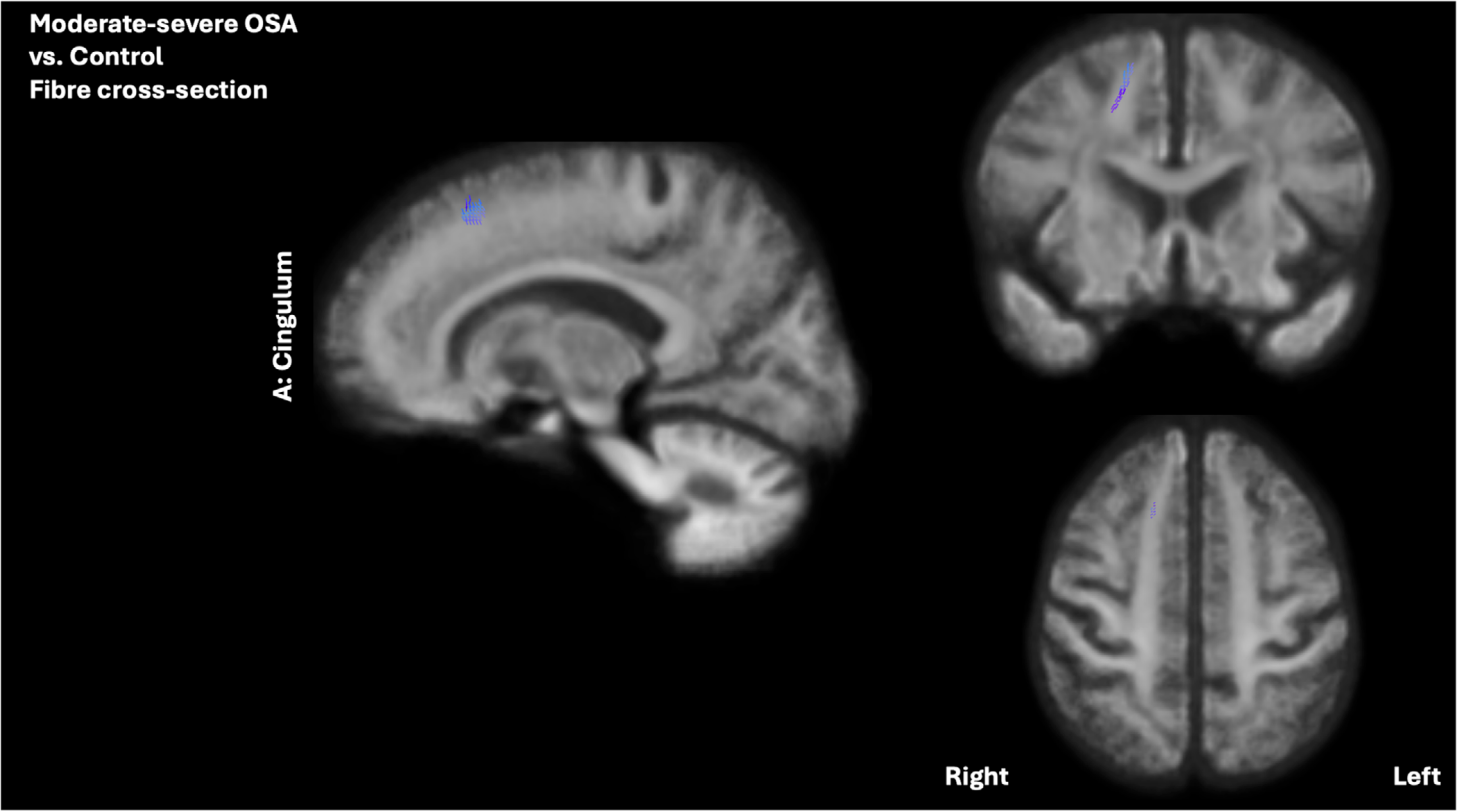
Fixel‐based comparison of white matter alterations between the moderate and severe OSA and control groups in older adults at‐risk of dementia, showing the significant fixels after FEW correction (*p* < 0.05). Results of fixel‐wise generalized linear models showing a decrease in Fiber cross‐section within the right cingulum tracts in moderate and severe OSA, compared to the control group. Each fixel is color‐coded according to its principal fiber orientation: red represents left‐right (*x*‐axis), green represents posterior‐anterior (*y*‐axis), and blue represents inferior‐superior (*z*‐axis) directions. OSA, obstructive sleep apnea.

As shown in Figure 3, participants with moderate and severe OSA exhibited widespread increases in log FC compared to the control group, specifically in the right cingulum (136 significant fixels). This was accompanied by a slight reduction in FDC within the left cingulum (14 significant fixels). For the fornix, the moderate and severe OSA group exhibited a decrease in FD (62 and 23 significant fixels in the left and right, respectively) and an increase in log FC (15 and 609 significant fixels in the left and right, respectively) compared to the control group, as depicted in Figure 2B.

After controlling for BMI, the previously significant fixel metrics in the cingulum were no longer significant. However, there was a small increase in FD within the right UF (one significant fixel).

Similarly, the adjustment for hypertension eliminated the cingulum's significance in the GLM. Nonetheless, the reduction in FD in the left fornix and increase in log FC in the right fornix remained significant (157 and 457 significant fixels, respectively).

### 3.3 Within‐group associations between alterations in FBA metrics and learning and memory performance

To investigate whether changes in white matter were linked to memory, within‐group analyses were conducted separately for the mild OSA and moderate and severe OSA groups. In the mild OSA group, none of the FD, FC, or FDC alterations were associated with learning or memory performance (all *p*_FWE _> 0.05).

**FIGURE 4:**
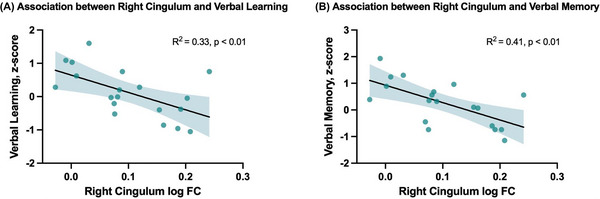
Scatterplot demonstrating the association between verbal (A) learning and (B) memory and the average log FC values in the right cingulum in those with moderate and severe OSA (*n* = 19). The average right cingulum values were restricted to the region that showed a significant difference between controls and moderate and severe OSA. Verbal learning and memory were assessed by the RAVLT. FC, fiber cross‐section; OSA, obstructive sleep apnea; RAVLT, Rey Auditory Verbal Learning Test.

In the moderate and severe OSA group, a lower log FC in the right cingulum was significantly linked to poorer verbal learning (*p*_FWE _< 0.05) and memory (*p*_FWE _< 0.05). These associations remained significant after controlling for hippocampal volume (*p*_FWE _< 0.05). Both findings were further confirmed using linear regression analyses conducted within the moderate and severe group, as illustrated in Figure 4. Mean log FC values were extracted from the portion of the cingulum tract that demonstrated a significant between‐group difference, as shown in Figure 3. They were significantly associated with verbal learning (*β* = −0.58, *p* = 0.010, *R* ^2^ = 0.33) and memory (*β* = −0.64, *p* = 0.003, *R* ^2^ = 0.41). The linear regression analyses were repeated within the control group, and the mean log FC values of the right cingulum predicted neither verbal learning nor memory (see *Supplementary Table* *1*). Lastly, the FD and FDC were not associated with learning or memory in the moderate and severe group (all *p*_FWE _> 0.05).

## 4 DISCUSSION

To our knowledge, this is the first study that utilized FBA to investigate the impact of OSA on white matter microstructure in older adults with both subjective and objective cognitive impairment. Consistent with our predictions, participants with mild OSA showed widespread alterations in FBA metrics within the ATR, UF, and fornix compared with controls. However, contrary to expectations, no significant differences were detected in the cingulum for this group. In contrast, participants with moderate and severe OSA exhibited localized changes, including increased FC in the right cingulum, decreased FDC in the left cingulum, and altered FD and FC within the fornix. The effects on the fornix were evident in fewer fixels than those observed in the mild group. Finally, while alterations in the mild OSA group were not associated with learning or memory, the increased FC in the right cingulum in the moderate and severe group was associated with poorer learning and memory performance, independent of hippocampal volume. Together, these findings indicated that OSA‐related white matter alterations are region and severity‐specific.

The relationship between OSA severity and white matter microarchitecture in older adults in a memory clinic did not exhibit a linear dose–response association. More specifically, mild OSA had more extensive white matter changes than those in the moderate and severe OSA group. Although this finding may initially seem counterintuitive, our results align with a previous study of a younger typical OSA sample^45^ that used traditional DTI metrics, which similarly observed more widespread white matter skeleton disruptions in mild OSA relative to moderate and severe cases. Additionally, a tract‐specific study by the same research group identified significant associations between hypoxemia and alterations specifically within the fornix in MCI but not in cognitively intact participants.^20^ The study also did not observe significant changes in the cingulum or UF.

Competing pathophysiological mechanisms may explain the nonlinear dose–response association, as axonal loss from neurodegeneration or injury can decrease FBA metrics, while neuroinflammation and gliosis may increase them.^46^ Furthermore, we observed an increase in FC in the right cingulum in the moderate and severe OSA group, compared to the control group. Although it was expected that OSA would be linked to decreased FC, our findings are aligned with previous research that observed that older adults with subjective cognitive decline exhibited enlarged FC in the superior longitudinal fasciculus relative to controls.^47^ Notably, this enlargement was associated with decreased plasma beta‐amyloid (Aβ), indicating that elevated amyloid burden is associated with increased FC values. Rodent studies have shown that extracellular Aβ can cause axonal swelling.^48^ The cingulum is particularly susceptible to Aβ aggregation,^49^ linked to Aβ burden in OSA,^50^ as well as contributing to memory impairment in AD.^51^ Although increased diffusion metrics have been suspected to be linked to vasogenic oedema,^52^ our use of multi‐tissue constrained spherical deconvolution accounted for cerebrospinal fluid (CSF) ‐like free‐water,^53^ minimizing its influence. Therefore, the increased FC within the right cingulum may reflect axonal swelling associated with amyloid deposition, a hypothesis warranting further investigation.

This is reinforced by the differential impact of adjusting for other vascular risk factors such as BMI and hypertension across OSA severity groups. In individuals with moderate and severe OSA, the attenuation of white matter associations suggests that microstructural alterations in this group may be more closely linked to shared vascular and cardiometabolic pathways. This is concordant with the cardiovascular literature demonstrating the interconnected associations among OSA, BMI, and hypertension.^54^ Specifically, OSA has a strong association with BMI due to increased adipose disposition,^55^ while moderate and severe OSA has been linked to an increased risk of hypertension.^56^ In contrast, the persistence of white matter alterations in mild OSA after controlling for these factors supports the possibility that the microstructural findings may be due to a nonvascular mechanism. These exploratory findings should be interpreted cautiously, as the moderate and severe subgroup was smaller and adjustment may further reduce statistical power. Future larger studies are necessary to further validate these preliminary and exploratory findings.

Nonetheless, the microscopic changes in the cingulum may be the most pertinent from a cognitive perspective. Notably, they were moderately associated with learning and memory, even after controlling for hippocampal volume. Previous work has shown that the cingulum and the left hippocampal volume independently predicted episodic memory performance.^17^ Building on this evidence, our novel findings suggested that microscopic white matter changes in the cingulum at least partly underpinned the compromised learning and memory performance observed in older adults with OSA, in addition to the established contributions of hippocampal volume.^3^

Our study expanded on previous research by demonstrating significant involvement not only of the fornix but also the cingulum, UF, and ATR, thus advancing our understanding of the potential tract‐specific vulnerabilities in older adults with subjective and objective cognitive impairment and OSA. The observed differences in findings are likely attributable to our use of the fixel‐based methods, which, compared to traditional diffusion metrics, offered greater specificity and were less susceptible to inaccuracies caused by crossing fibers.^22^ Additionally, our analysis employed fixel‐level comparisons rather than averaging metrics across entire tracts, which enabled the detection of localized tract‐specific alterations. Indeed, our study replicated previously reported white matter changes in the fornix; we found that alterations extended across nearly the entire tract, whereas in other tracts, differences were confined to specific segments. This pattern may explain discrepancies with earlier studies,^57^ which likely lacked sensitivity to detect tract‐specific effects, as tract‐averaged metrics can mask local differences.

While there has been a dearth of research directly investigating OSA effects on the specific white matter tracts assessed here, these tracts are well‐established components of memory networks. The ATR links the hippocampus and to the anterior cingulate cortex, supporting the encoding of novel stimuli.^58^ The cingulum bundle interconnects the frontal, parietal, and medial temporal regions, which are involved in executive control and episodic memory.^59^ Its disruption may affect the cholinergic limbic pathway,^60^ which supports memory, learning, brain homeostasis, and neural plasticity.^61^ The UF connecting the anterior temporal lobe with the orbitofrontal cortex has previously been implicated in OSA‐related memory impairments; however, we did not observe a significant association between UF integrity and memory performance in our sample. Widespread alterations were also observed in the fornix, a key hippocampal output tract essential for memory consolidation and retrieval.

These findings have several important implications. The observation that mild OSA shows greater white matter disruption than moderate and severe OSA challenges the conventional dose‐response assumption. This suggests that mild OSA may represent a critical window for intervention to prevent diffuse white matter alterations before cognitive consequences emerge. In contrast, moderate and severe OSA showed more focal cingulum alterations linked to learning and memory deficits in this memory clinic sample of SCI and MCI. Early detection and treatment of OSA within memory clinics may offer a valuable strategy for preventing or reversing white matter changes, which are crucial for learning and memory, and reducing long‐term dementia risk. While there is not yet a robust evidence base for widespread screening and treatment of OSA to prevent dementia,^62^ screening and detection of OSA in the memory clinic should be considered,^1^, ^63^ particularly if early treatment for OSA could improve memory.^64^

This study offers novel insights into the mechanisms by which OSA may compromise brain integrity and cognition in older adults at a heightened risk for dementia. It may also inform future research. For instance, clinical trials could stratify participants by OSA severity to clarify whether early intervention for mild versus moderate and severe OSA confers greater neuroprotective benefits. Complementary indices, such as hypoxemia during REM sleep^3^ or hypoxic burden,^65^ may further refine characterization of hypoxemia‐related pathology and longitudinal studies could tract both white matter alterations and memory decline across OSA severity. Finally, the hypothesized competing mechanisms in moderate and severe OSA that may dilute white matter microstructural changes remain untested and are critical to resolving current inconsistencies in the literature.

The results of this study should be interpreted in the context of some limitations. The cross‐sectional design of the current study is insufficient to make definitive causal inferences. Furthermore, while the current study investigated structural abnormalities such as white matter microstructure and hippocampal volume, our sample was not phenotype with AD biomarkers (e.g., Aβ and pathological tau) and did not examine patterns within apolipoprotein (Apo) E4 subgroups. As noted, we used ODI as the primary measure of OSA severity and did not include alternative indices, such as hypoxic burden, because a legacy sleep system in our dataset lacked the data necessary for retrospective calculation. Nonetheless, ODI is a clinically well‐validated marker of intermittent hypoxemia, relevant to our outcomes and widely available across historical and current sleep systems, supporting its appropriateness and interpretability. A further limitation is the generalizability of our findings. The cohort is predominantly well‐educated and English‐speaking, which may not reflect the sociocultural and linguistic diversity of broader populations.

In conclusion, utilizing FBA enabled the detection of subtle, specific white matter disruptions. Our findings delineated an additional mechanism through which OSA may contribute to dementia pathophysiology. Together with prior studies, our findings highlighted the importance of early recognition and management of OSA in memory clinic populations.

## CONFLICT OF INTEREST STATEMENT

Prof Sharon Naismith has received consulting fees from Eisai and Roche Pharmaceuticals. All other authors have no conflicts of interest to declare.

## CONSENT STATEMENT

All participants provided written informed consent prior to any study procedures. The study was approved by the University of Sydney Human Research Ethics Committee and conducted in accordance with the ethical standards in the Declaration of Helsinki.

## Supporting information



Supporting information

Supporting information
