# Supplementary material for: Microscopic white matter changes in the cingulum contribute to memory impairment among older adults with obstructive sleep apnea in the memory clinic
Source: Alzheimers Dement. 2026 Feb 13;22(2):e71197. doi: 10.1002/alz.71197 (PMC12902798; doi:10.1002/alz.71197)
Supplement: Supplementary file 2 — Supporting information [file ALZ-22-e71197-s002.docx]

Supplementary Materials

| Supplementary Table 1. Linear Regression analyses within control group | | | | |
| --- | --- | --- | --- | --- |
| Verbal Learning | | | | |
| Parameter | Estimate | Std. Error | 95% CI | p-value |
| Slope (β₁) | -1.692 | 2.883 | -7.749 - 4.364 | 0.564 |
| Intercept (β₀) | -0.055 | 0.302 | -0.690 - 0.579 | — |
| R-squared | 0.0188 | — | — | — |
| F(1, 18) | 0.3447 | — | — | 0.564 |
|  |  |  |  |  |
| Verbal Memory | | | | |
| Parameter | Estimate | Std. Error | 95% CI | p-value |
| Slope (β₁) | -1.050 | 3.342 | -8.072 - 5.97 | 0.757 |
| Intercept (β₀) | -0.065 | 0.350 | -0.801 - 0.670 | — |
| R-squared | 0.0055 | — | — | — |
| F(1, 18) | 0.0987 | — | — | 0.757 |
|  | | | | |
